# Supplementary material for: Circular RNAs arising from synaptic host genes during human neuronal differentiation are modulated by SFPQ RNA-binding protein
Source: BMC Biol. 2023 May 26;21:127. doi: 10.1186/s12915-023-01627-w (PMC10224606; doi:10.1186/s12915-023-01627-w)
Supplement: Supplementary file 1 — Additional file 1: Figure S1. Validation of circRNA expression. Figure S2. Validation of circRNA exon retention. Figure S3. Pathway enrichment among differentially expressed circRNAs. Figure S4. Pathway enrichment among differentially expressed linear RNAs. Figure S5. Log2 fold-change values of top increased circRNAs and their corresponding linear host RNAs. Figure S6. Variant analysis in circRNA exons. Table S5. Source studies for compilation de novo variants identified in individuals with Schizophrenia or Schizoaffective disorder. Figure S7. SFPQ and TIAL1 target detection and siRNA knockdown. Figure S8. Extended expression profile of SFPQ and identified circRNA targets in NES differentiation. Figure S9. In Situ detection of circRNAs during NES differentiation. Figure S10. Analysis of introns from circRNA host genes. Table S8. Primer sequences. [file 12915_2023_1627_MOESM1_ESM.pdf]

SUPPLEMENTARY INFORMATION

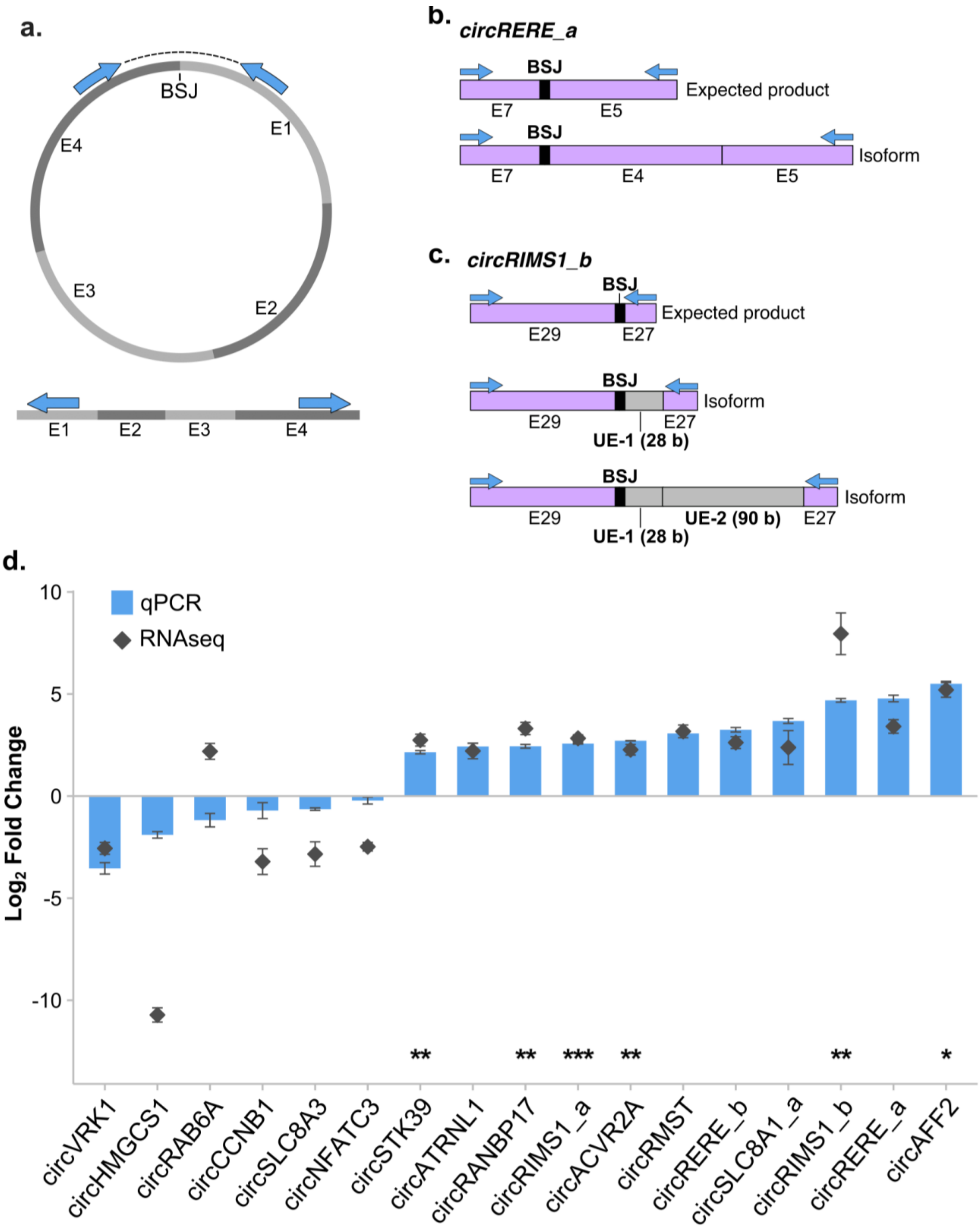

Figure S1. Validation of circRNA expression**. a.** Schematic representation of junction primer design (blue arrows) in relation to the circular and linear forms of RNA. **b,c.** Schematic representation of Sanger alignment from isoforms amplified by junction primers for *circRERE_a* (b) and *circRIMS1_b* (c). BSJ = Back Spliced Junction, E = Exon. UE = Unannotated Exon. **d.** Log fold-change of differentially expressed circRNAs from qPCR and RNAseq data, D28 vs NES (*n* = 3), two-sample t-tests, Bonferroni corrected. Error bars represent SEM, ** = p-adj <0.05, ** = p-adj <0.01, *** = p-adj <0.001.

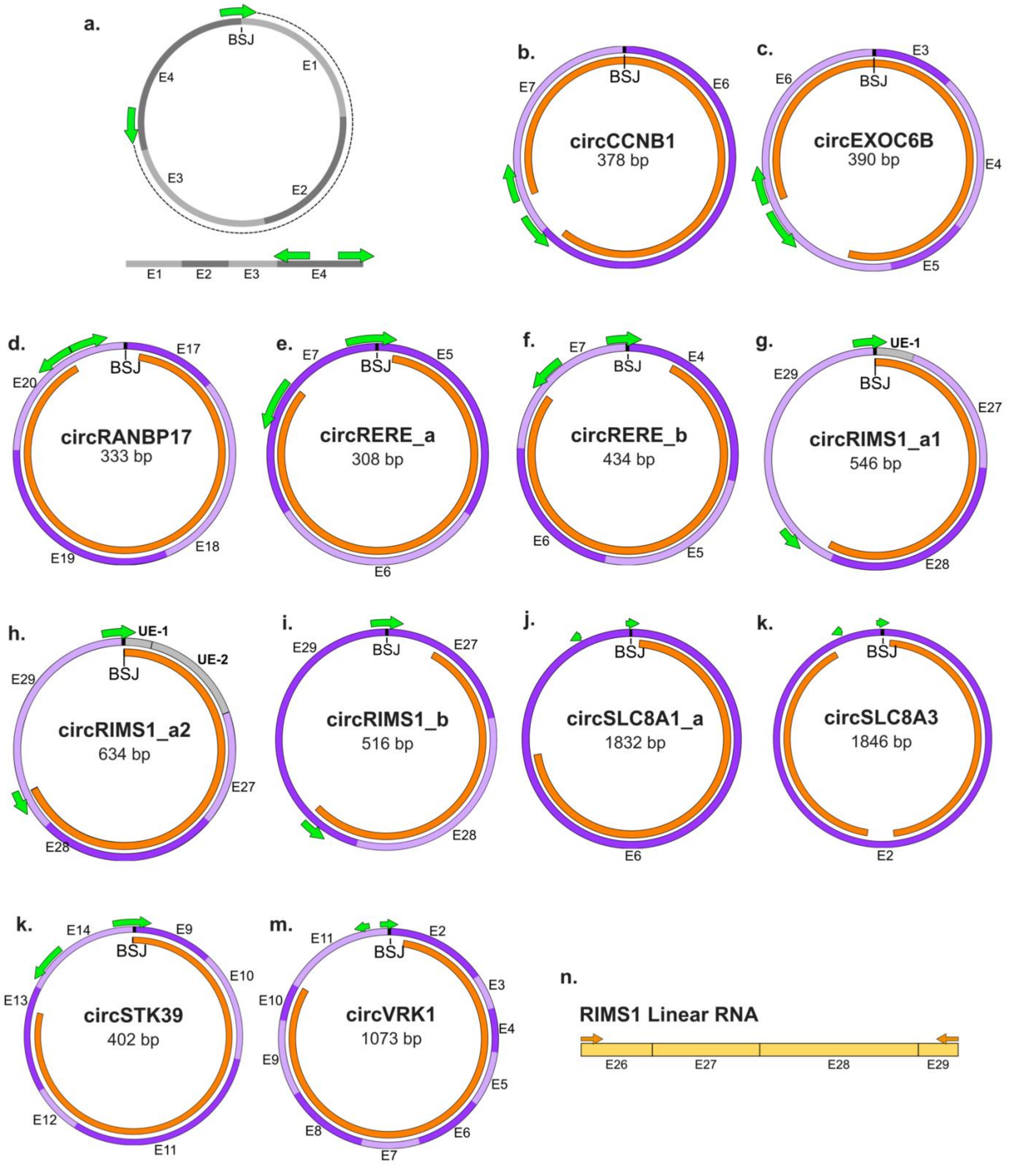

Figure S2. Validation of circRNA exon retention. **a.** Schematic representation of circular primer design (green arrows) in relation to the circular and linear forms of RNA. **b-m.** Schematic representations of predicted circRNA exon annotations and sanger sequencing alignments. Exons are shown in purple, green arrows indicate primer location and sanger sequencing product alignment is shown in orange. BSJ = Back spliced Junction, E = Exon, UE = Unannotated Exon. **n.** Schematic representation of Sanger sequencing results from PCR targeting exons 26 – 29 of the linear RNA form of RIMS1, flanking the unannotated exons detected in circular RIMS1 isoforms.

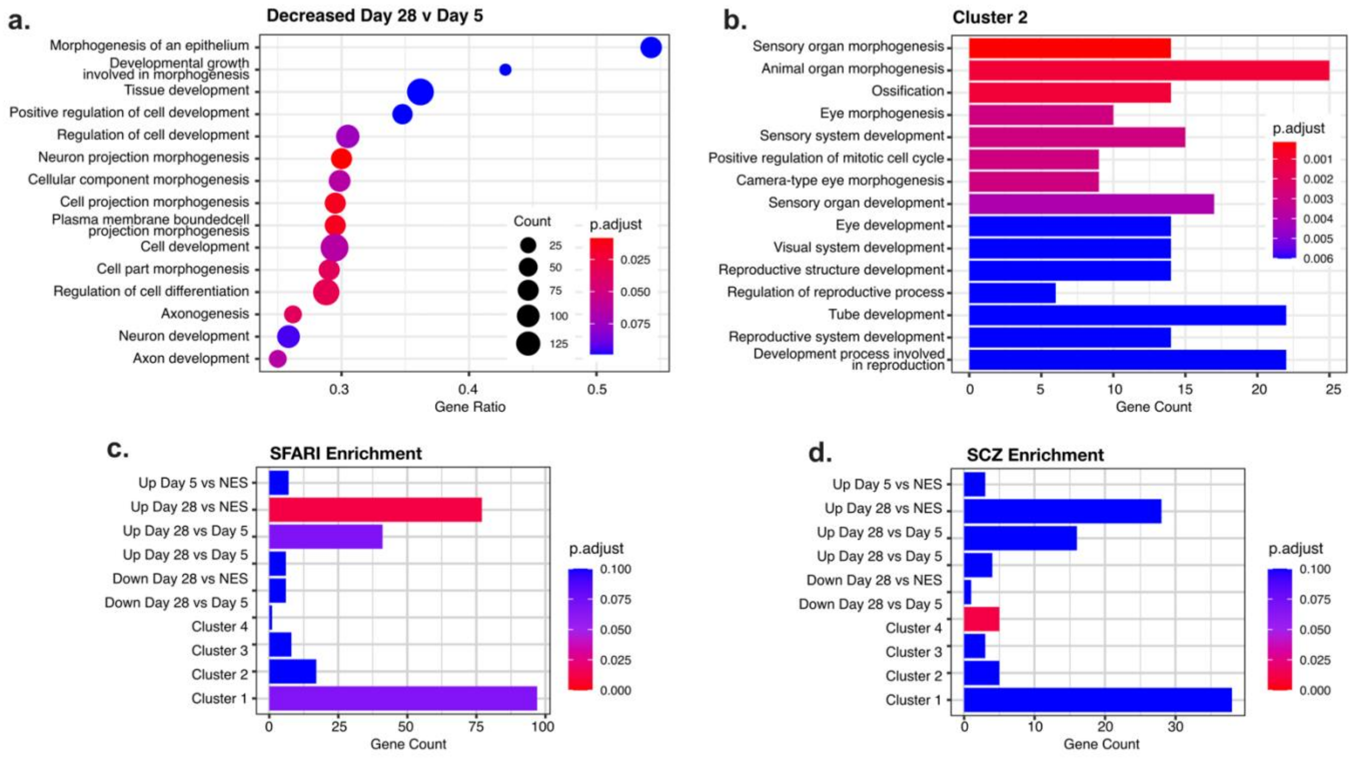

Figure S3. Pathway enrichment among host genes of differentially expressed circRNAs. **a.** GSEA of gene ontology - biological process terms among host genes of circRNAs decreased at Day 28 vs Day 5. **b.** Most highly enriched gene ontology - biological process terms among Cluster 2 circRNA host genes. **c.** Enrichment of SFARI risk genes among DEcirc host gene sets. **d.** Enrichment of Schizophrenia (SCZ) risk genes among DEcirc host gene sets.

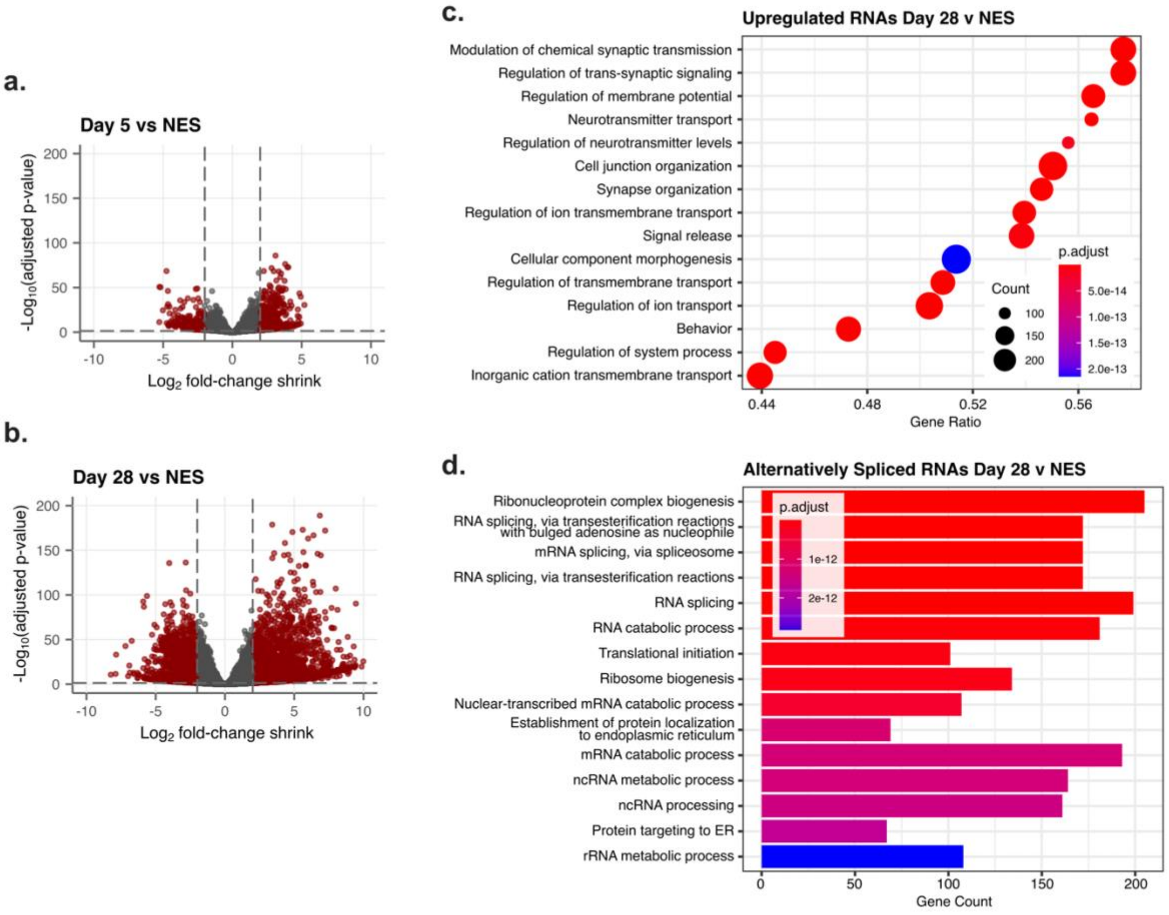

Figure S4. Pathway enrichment among differentially expressed linear RNAs. **a,b**. Volcano plots of differentially expressed RNAs at D5 and D28 of differentiation compared to NES. **c.** GSEA of gene ontology - biological process terms among RNAs increased at D28 vs NES. **d.** Most highly enriched gene ontology - biological process terms among RNAs that were alternatively spliced at D28 vs NES.

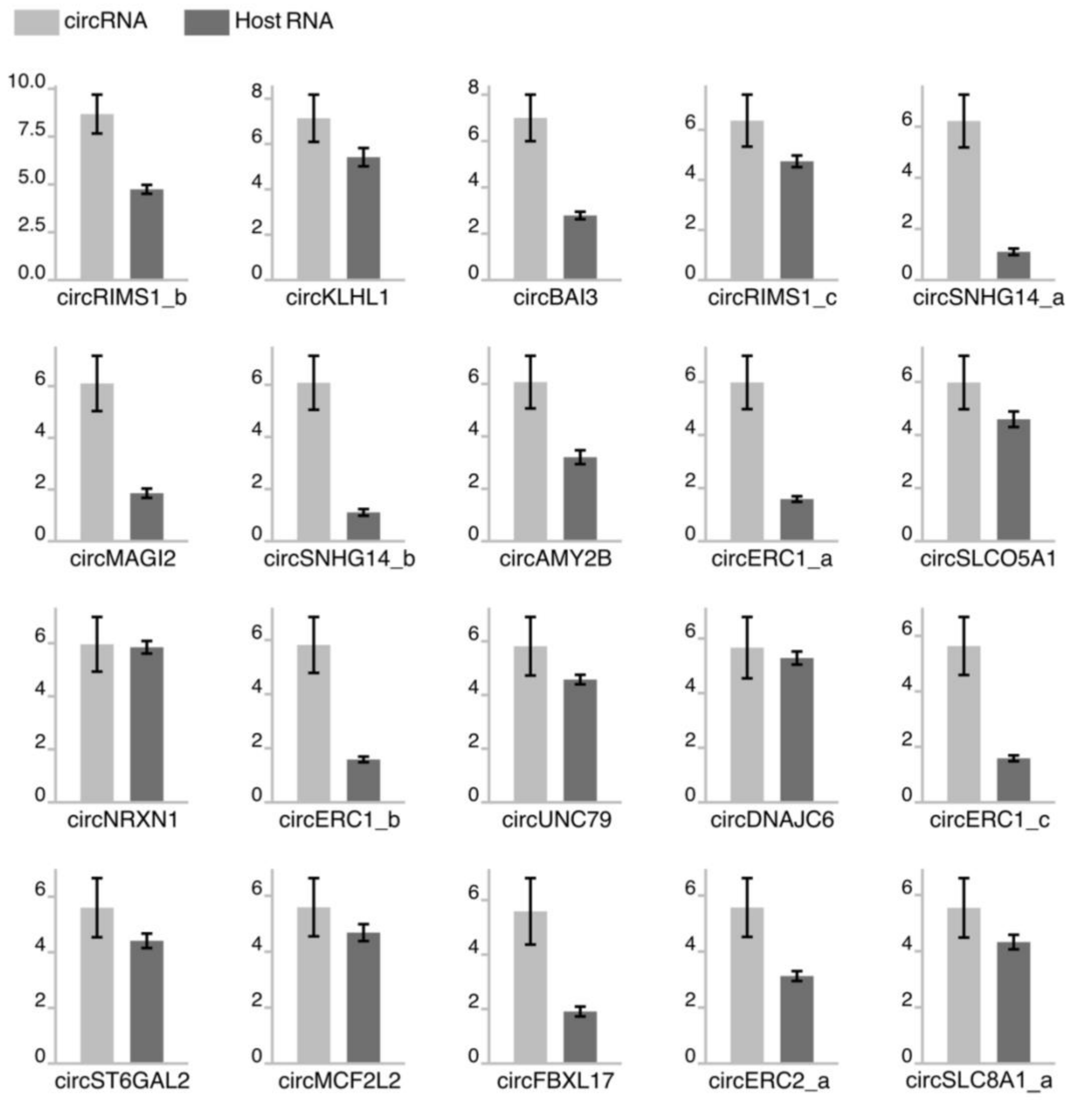

Figure S5. Log2 fold-change values of top increased circRNAs and their corresponding linear host RNAs (D28 vs NES).

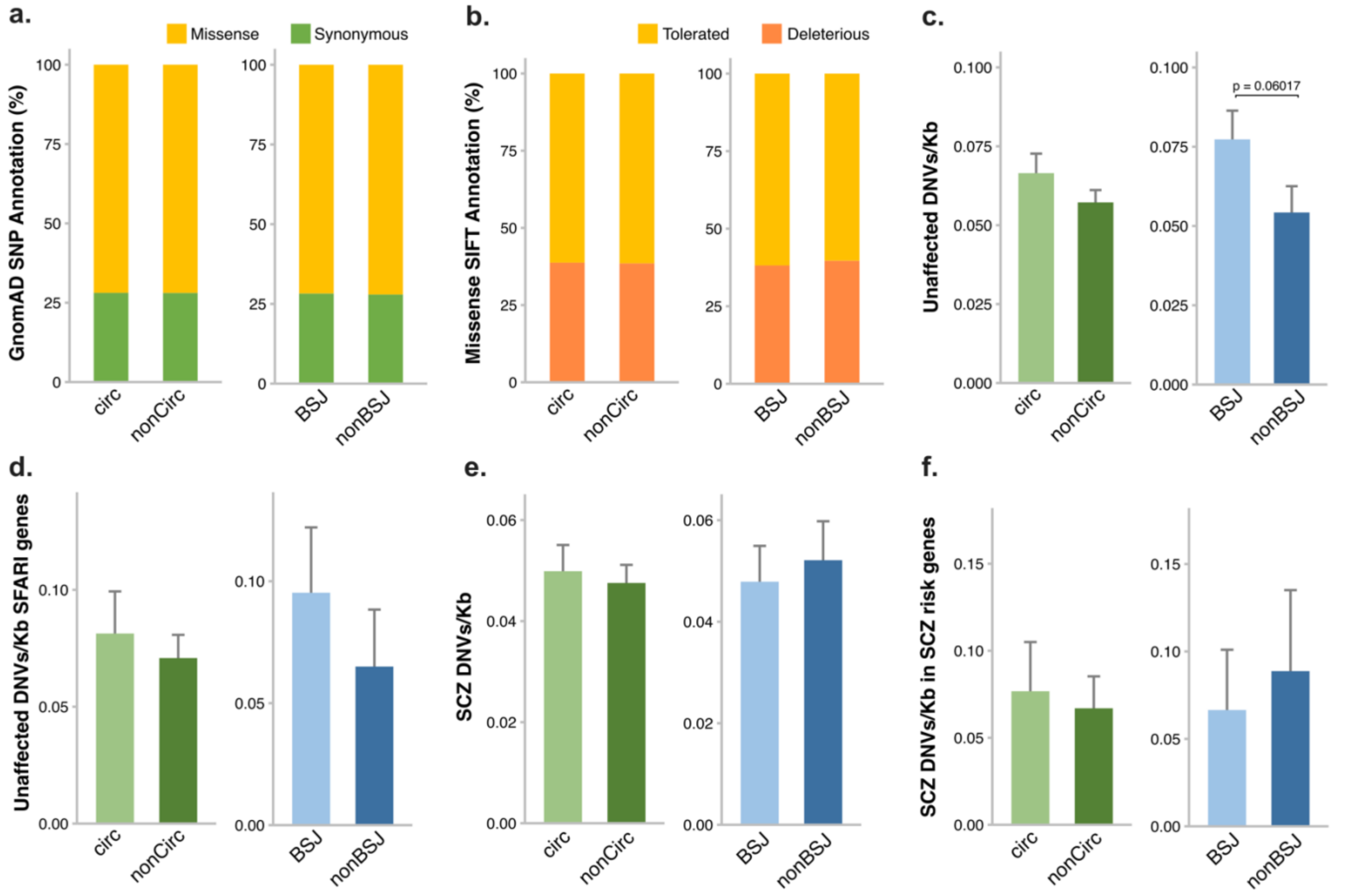

Figure S6. Variant analysis in circRNA exons**. a.** Proportion of missense or synonymous SNPs among gnomAD variants. **b.** Proportion of gnomAD missense SNPs classified as 'Tolerated' or 'Deleterious' by SIFT prediction. **c.** Frequency of rare *de novo* variants/Kb in unaffected sibling controls. **d.** Frequency of rare *de novo* variants/Kb in unaffected sibling controls in exons of circRNAs arising from ASD-associated SFARI host genes. **e.** Rate of Schizophrenia (SCZ) *de novo* variants per kilobase in exons of circRNAs. **f.** Rate of SCZ *de novo* variants per kilobase in exons of circRNAs arising from Schizophrenia risk host genes. DNVs = *de novo* variants.

Table S5. Source studies for compilation of *de novo* variants identified in individuals with Schizophrenia or Schizoaffective disorder.

	Authors (Year)	Journal	DOI	Data Type	Gene association with Schizophrenia
**[1]**	Ambalavanan *et al* (2015)	*Eur. J. Hum. Genet.*	DOI: 10.1038/ejhg.2015.218	WES	Damaging or missense *de novo* mutations in cases.
**[2]**	Fromer *et al* (2014)	*Nature*	DOI: 10.1038/nature12929	WES	*De novo* mutations in schizophrenia probands (no missense predictions).
**[3]**	Girard *et al* (2011)	*Nat. Genetics*	DOI: 10.1038/ng.886	WES	Damaging or missense *de novo* mutations in cases.
**[4]**	Guipponi *et al* (2014)	*PLoS ONE*	DOI: 10.1371/journal.pone.0112745	WES	Damaging or missense *de novo* mutations in cases.
**[5]**	Gulsuner *et al* (2013)	*Cell*	DOI: 10.1016/j.cell.2013.06.049	WES	Damaging or missense *de novo* mutations in cases.
**[6]**	McCarthy *et al* (2014)	*Mol. Psychiatry*	DOI: 10.1038/mp.2014.29	WES	Damaging or missense *de novo* mutations in cases.
**[7]**	Takata *et al* (2014)	*Neuron*	DOI: 10.1016/j.neuron.2014.04.043	WES	Loss-of-function *de novo* indel variants.
**[8]**	Wang *et al* (2015)	*Scientific Reports*	DOI: 10.1038/srep18209	WES	Nonsynonymous *de novo* variants.

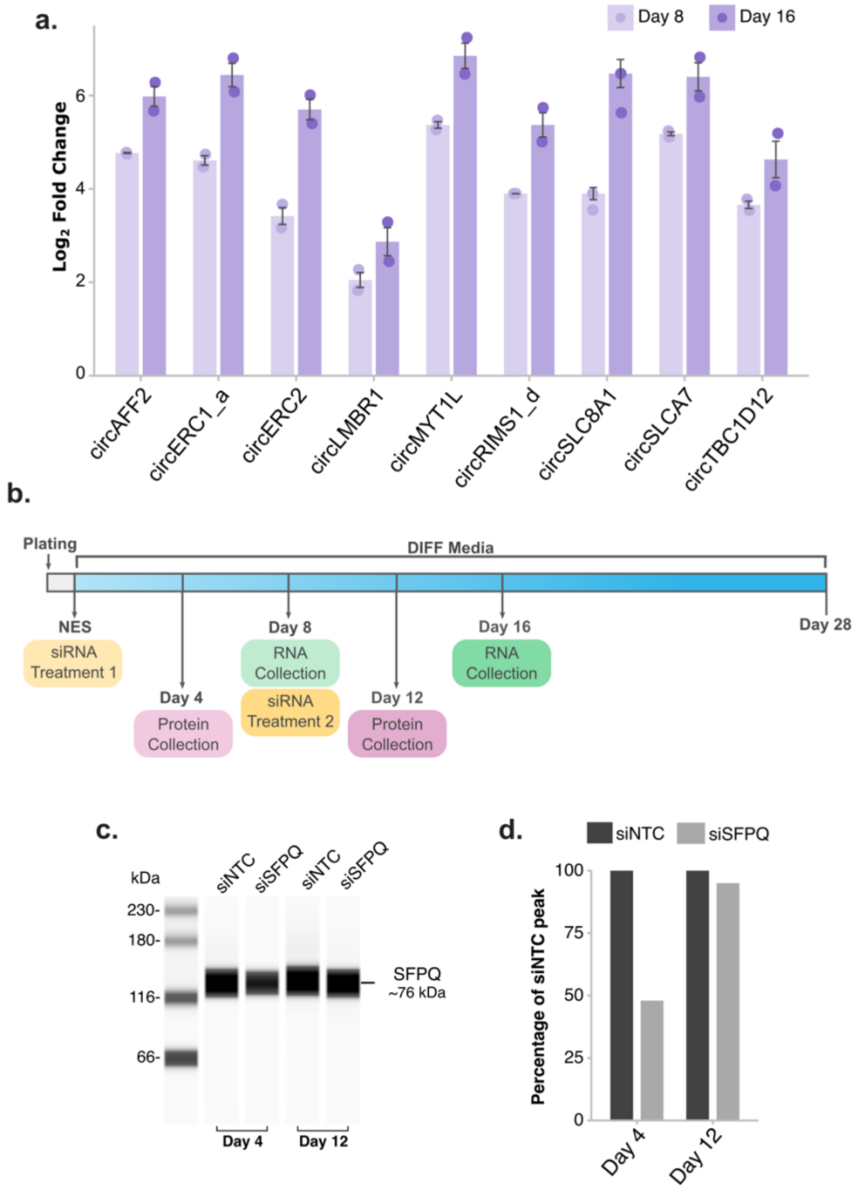

Figure S7. SFPQ and TIAL1 target detection and siRNA knockdown**. a.** Detection of circRNA targets during differentiation by qPCR, (n = 2). **b.** Schematic of siRNA knockdown experiment timeline and sample collection. **c.** Detection of SFPQ protein following siRNA treatment by WES. **d.** Quantification of SFPQ WES, normalised to total protein.

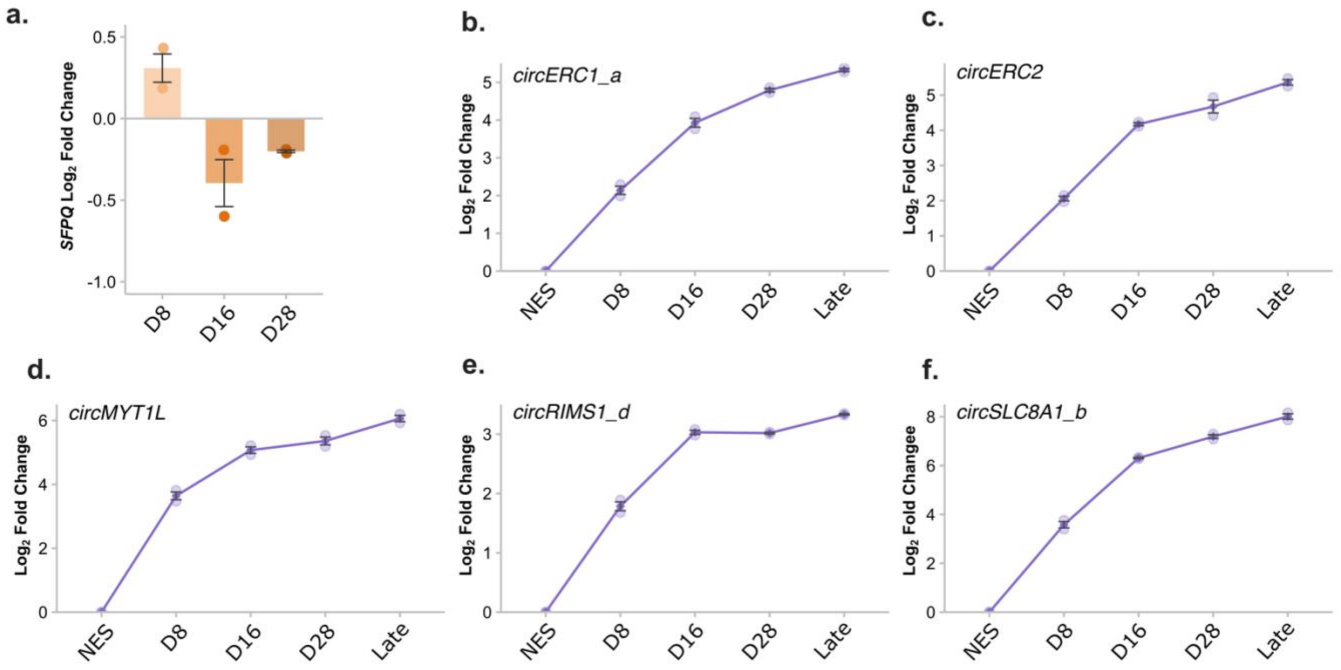

Figure S8. Extended expression profile of SFPQ and identified circRNA targets in NES differentiation. **a.** SFPQ expression at days 8, 16 and 28 of differentiation relative to NES cells (n = 2). **b-f.** SFPQ target circRNA expression detected by qPCR from NES to samples collected after either 40 or 50 days of differentiation (Late) (n = 2).

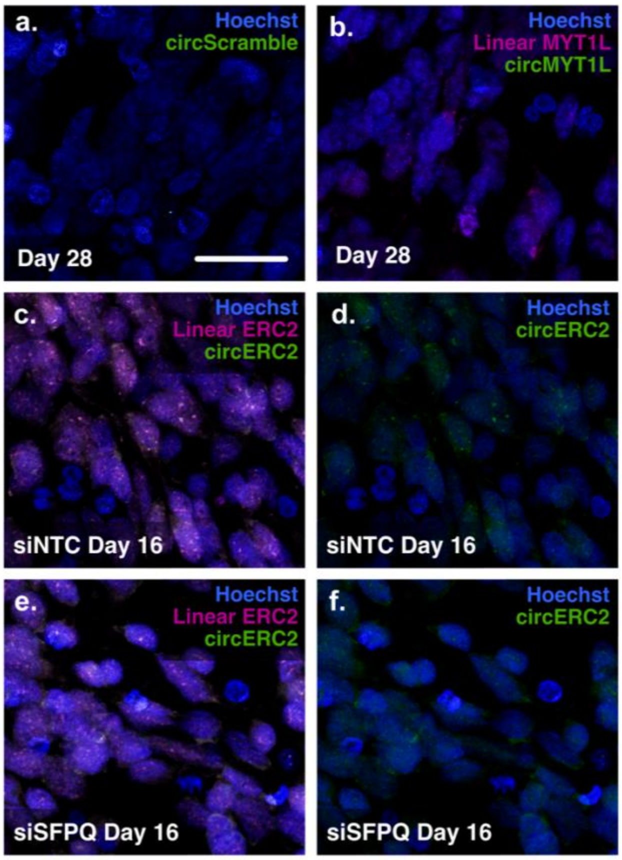

Figure S9. *In Situ* detection of circRNAs during NES differentiation. **a.** Signal from the negative control circScramble probe at D28. **b.** Detection of *circMYT1L* and its linear counterpart at D28. **c,d.** Detection of *circERC2* and its linear counterpart at D16 following no-target siRNA control treatment (siNTC). **e,f.** Detection of *circERC2* and its linear isoform at D16 following siRNA knockdown of SFPQ (siSFPQ). Blue = Hoechst nuclear counter stain, magenta = linear probes, green = circ probes. Scale bar in a = 25 µm, applies to all images, images are representative of staining from biological replicates (*n* = 3).

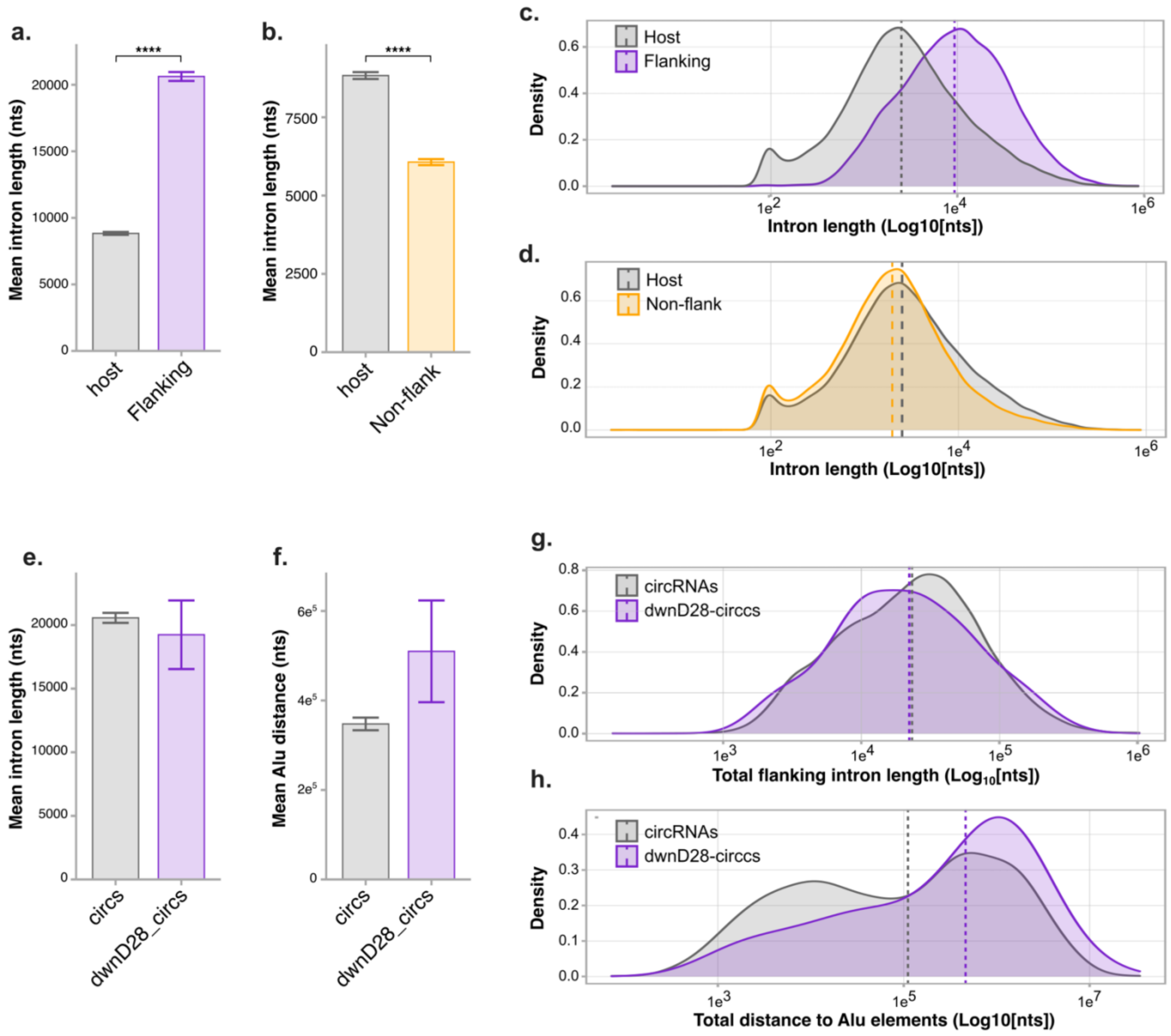

Figure S10. Analysis of introns from circRNA host genes. **a.** Mean intron length of circRNA flanking introns compared to all introns in circRNA host genes. **e.** Mean length of host introns that do not flank circRNAs compared to all introns in circRNA host genes. **c.** Density plot of length of circRNA flanking introns compared to all introns in circRNA host genes. **d.** Density plot of length of host introns that do not flank circRNAs compared to all introns in circRNA host genes. **e-f.** Analysis of introns flanking circRNAs decreased at D28vNES compared to all circRNAs. **e.** Mean intron length of flanking introns. **f.** Mean distance to IAE from BSJ. **f.** Density plot of intron length totalled from both flanking introns. **g.** Density plot of IAE distance totalled from Alu element distance on both flanking introns Error bars in a, b, e and f represent standard error, dashed lines in c, d, g and h indicate median values. Two sample Z-test, **** = $p < 0.0001$.

Table S8. Primer sequences.

Target	Forward primer 5' > 3'	Reverse primer 5' > 3'
	Divergent BSJ Primers	
circACVR2A	ATTGCGGGGATTGTCATTTG	ACCAGTTTGATTGGTTCTGTC
circAFF2	GTCATTTGGAACACTCTTGG	GACTCGGTTGGCAAGTG
circATRNL1	CATTCTATGTGTACGTCAGCAAC	TTTATGGCAGTATGGTGGCGAT
circCCNB1	GCACTGAAAATTCTGGATAATGGTG	TTGCTCGACATCAACCTCTCCA
circCSNK1G3	GAATATGTGGCAATTAAGTTGGAGC	ATGTGAGCTGATATTGATAGAGAGC
circERC1_a	TCTCTTGGAGCTTTCGTCCTC	GGCCATCAGTAACTCCTCCAC
circERC2	TTGGCCAACCTCCGGATTG	CTGCGCACTTCTTCTAGTAACTG
circEXOC6B	GGAGAGCATCCGCAAACATTC	TAGTCGACACTGCTTCAGCTC
circHMGCS1	TCAGCAATTAGCAGGGAAGAG	TGCATATGTGTCCCACGAAG
circLMBR1	GGTCAGTTGCTAGTGAAGCC	TAAAAGCAAAACAGCCCCAGC
circMYT1L	AGCCTAAATCCAGTGACAGCC	GCCACCAGTTCATCGTAATTGTC
circNFATC3	TAGCCGAGGGGCAGTAAAAG	GTGGTAAGCAAAGTGGTGTGG
circPTK2	CAGCTAGTGACGTATGGATGTT	GACGCATTGTTAAGGCTTCTTG
circRAB6A	AGGGAGAGAGGAAAGCCAAAGA	GTTCCTGACCCGCAGTATCCC
circRANBP17	TCGAGGGATTGCCTTTGCAC	TTTTAGCATGAATTTCACAGCATCT
circRERE_a	AGAGTTTAAAGCCCGAGTGGA	TCGATCCTGAACCAAATGCTGA
circRERE_b	AGAGTTTAAAGCCCGAGTGGA	GGTCACACAAAGCAGGAGTTG
circRIMS1_a	AGCAGGTGGAAAGAAACGGA	AGCTTGCTTTTGTGGAAGAGTTA
circRIMS1_b	AGCAGGTGGAAAGAAACGGA	CGCTCCTCCACTACTAAGC
circRIMS1_d	TCAGTGATGTTTCCGCCATTTC	TTGTTCGCTCCTCCACTACT
circRMST	TTGCTCAAGGTGGAACGACA	TTGCTCAAGGTGGAACGACA
circRTN4	ACGATAAAGGAACTCAGGCG	AATAGGCTGGCACCAAACAC
circSLC4A7	GATGAAATGGCCAAAACTGCAC	TCCAAGTTTCCAGGAGCAGAC
circSLC8A1_a	GTGGAGGGGAGGATTTTGAG	TCCCATTGAAAAGGTGGGTGAA
circSLC8A1_b	TGCACCATTGGCCTGAAAG	GGCGGGGCTCTCCAATCTC
circSLC8A3	GACAGTAGAAGGGACAGCCA	TTGCAGCACCAGTTGTCCTC
circSTK39	AGCTTCTTCTTGTGCCGTGA	TCTGGTGTTCTTGTAAGCAGC
circTBC1D12	GGGGAGGACCTGTAAACCAC	TCCAACCAGGAGCACTATGG
circVRK1	TGGACCTCAGTGTTGTGGAG	CTCCAACTGCAAATTGTTCTGC
circZNF91	AGAAGTGTAATTACTGTCAAACGAC	TGCTCTGGCCAAAAGTCTTGA

	Circular divergent primers	
circATRNL1	GCTGGAACAATATCTGGGGAAGA	TTGAACCGACAGACCACGTAA
circCCNB1	GGCCAAATACCTGATGGAACTAACT	TTGCTCGACATCAACCTCTCCA
circEXOC6B	GGTGGACAACATCCCCAAGC	TTGCAGAATCGATAGTGGCTTAC
circNFATC3	CAGGGGAAGTGTGACAGAAGATAC	CAGGTGAAGGAACAGGTGAGTG
circPTK2	GTCTGGATAATCATGGAGCTGTG	TGATGACTCCAATCAGCTTCAC
circRAB6A	CGTAGCCTCATTCCCAGTTACATCC	GTTCCTGACCCGCAGTATCCC
circRANBP17	CAAAGACCAGCTACACCATGC	TGTTCAGTGCAAAGGCAATCC
circRERE_a	ACCCTGAGACAAGAGTAAGAG	TCGGGCTTTAAACTCTCTAGCA
circRERE_b	CCCTGAGACAAGGTCCACAA	TCGGGCTTTAAACTCTCTAGCA
circRIMS1_a	CAGCTTAGTCAAACAGAACAAG	GAAGTCCCCATCCGTCTG
circRIMS1_b	AGTCAAACAGCAAGCTTAGTAG	GAAGTCCCCATCCGTCTG
circSLC8A1_a	TGATGAAATTGTTAGGTTGTGACA	CATGATGCCAATGCTCTCAC
circSLC8A3	ATGATGAAACTGTGTCTCTGGC	CACCTGATGTCCGCAGAAC
circSTK39	TTGAGATTAAGGCCCACAGCA	TAGTCTTCATTAGCATTGGGTG
circVRK1	CAATAACAAAGTGAAAATGCCTCGTG	ATTCTCCACAACACTGAGGTC

	Linear Primers	
5SrRNA	CGCCCGATCTCGTCTGAT	GGTCTCCCATCCAAGTACTAACCA
AFF2	CCAGTGGTCAGCCAAACAAG	TGTCTTCCTTTGCAGATGGGT
CPSF6	GATTCCATGGCATATGGGGC	TGGAGTCTGTCCAGCTTTTAGG
ERC1	AATCTTCGGGCAGAGAGAAGG	AGAGGACGAAAGCTCCAAGAG
ERC2	AGCATGGCTGACAACTCACA	TTCTTTTTCGGCCAGGGACT
GAPDH	AAGGTGAAGGTCGGAGTCAAC	GGGGTCATTGATGGCAACAATA
LMBR1	CACAGTGATGGGTCAGTTGC	TTAGTCGTCTCTGGAGTGCTTC
MYT1L	TACGTCATGTTGGGGAAGCC	TTCTGCTGCGGATTCCTCTC
RIMS1	GGAGGAGCGAACAAGACAGA	GTTCGGGAAATGGCGGAAAC
RIMS1 exons 26-29	CAGACACATCGTTCAGCAGTCG	GAAGTCCCCATCCGTCTG
SFPQ	CCAGCATGGCACGTTTGAG	TGTCGTCTCATCAGATCTTGGC
SLC4A7	CTGAACGGATGCTTCAAGATGA	CTTCCACTGTGCAGTTTTGGC
SLC8A1	CTTGTGGTTGGGACTAACAGC	ACGTAATCGAAACAGGAGGGC
SPAG9	GCAGCTCATCACCCAGTACG	TCTTCAAGTCTGCTAATCTGGTCA
TBC1D12	ACATCCAAAATCATTCAGCAGGA	AATCAAGGCAGTGGTAGAAAGC
TIAL1	TGCGTCTGGGTTAACAGATCA	GGCTGCACTTTCATGGGTTG

Table S9. Probe designs for *in situ* hybridisation. Junction indicates which exon/exon junction the probe spans. Transcript ID = Ensembl transcript ID. Bases highlighted in red indicate bases with locked nucleic acid modifications.

Probe	Transcript ID	Junction	Sequence 5' – 3'	Length	Tm (°C)
circERC2	ENST00000288221	14/13	FAM-T**C**TTATTCT**G**ATCTTTCATAT**G**CTTCAT**C**TCCAGGAT**C**TCCTC**C**AG	46 b	70.4
Linear ERC2	ENST00000288221	14/15	Cy3-T**G**GCTGCAA**G**TAGTGCTT**C**CTGTTT**C**ATCTCCAG**G**ATCTCCTC**C**AG	46 b	75.2
circMYT1L	ENST00000428368	10/10	FAM-TGA**C**AATTCATTT**G**ATGGTCTT**C**ATAGTATGG**C**TTTTTGA**C**ATGGCTGTCA**C**TGG	55 b	73.1
Linear MYT1L	ENST00000428368	10/11	Cy3-CT**C**TTTCTTTT**C**TGTTCTT**G**AGGGAT**C**ATAGTATGG**C**TTTTTGACAT**G**GC	50 b	70.8
Scrambled	-	-	FAM-TGC**G**TTCTCCTTC**T**AACTCGTG**C**AACACTT**C**GATCTATA**G**TACT**C**T	46 b	70.3
